# Decreased miR-320a promotes invasion and metastasis of tumor budding cells in tongue squamous cell carcinoma

**DOI:** 10.18632/oncotarget.11612

**Published:** 2016-08-25

**Authors:** Nan Xie, Cheng Wang, Zehang Zhuang, Jinson Hou, Xiqiang Liu, Yue Wu, Haichao Liu, Hongzhang Huang

**Affiliations:** ^1^ Department of Oral Pathology, Guanghua School of Stomatology, Hospital of Stomatology, Sun Yat-sen University, Guangzhou, Guangdong 510055, China; ^2^ Department of Oral and Maxillofacial Surgery, Guanghua School of Stomatology, Hospital of Stomatology, Sun Yat-sen University, Guangzhou, Guangdong 510055, China; ^3^ Guangdong Provincial Key Laboratory of Stomatology, Sun Yat-sen University, Guangzhou, Guangdong 510055, China

**Keywords:** tongue squamous cell carcinoma, tumor budding, miR-320a, Suz12, invasion and metastasis

## Abstract

We aimed to determine the specific miRNA profile of tumor budding cells and investigate the potential role of miR-320a in invasion and metastasis of tongue squamous cell carcinoma (TSCC). We collected tumor budding cells and paired central tumor samples from five TSCC specimens with laser capture microdissection and examined the specimens using a miRNA microarray. The specific miRNA signature of tumor budding cells was identified. We found that miR-320a was dramatically decreased in tumor budding cells. Knockdown of miR-320a significantly enhanced migration and invasion of TSCC cell lines. Suz12 was shown to be a direct target of miR-320a. Similar results were also observed in nude mouse models. Multivariate analysis indicated that miR-320a was an independent prognostic factor. Kaplan–Meier analysis demonstrated that decreased miR-320a and high intensity of tumor budding were correlated with poor survival rate, especially in the subgroup with high-intensity tumor budding and low expression of miR-320a. We concluded that decreased expression of miR-320a could promote invasion and metastasis of tumor budding cells by targeting Suz12 in TSCC. A combination of tumor budding and miR-320a may serve as an index to identify an aggressive sub-population of TSCC cells with high metastatic potential.

## INTRODUCTION

TSCC is much more aggressive than other cancers of the oral cavity and shows rapid local invasion [1] and spread [2]. Despite the continuous advancements in treatment modalities, the survival rate of TSCC patients has not significantly improved in the past decades [3]. Metastasis is still a major challenge to clinical treatment [4]. It was reported that 20% to 40% of cT1/2N0 stage TSCC patients already had occult cervical metastasis [5]. Therefore, it is urgent to identify novel biomarkers of metastasis for TSCC treatment.

Tumor budding is a microscopic finding located in the stroma ahead of the tumor invasive front (TIF). It is defined as the presence of isolated single cancer cells or small clusters of cancer cells (less than five cells). These budding cells indicate that the cells undergoing epithelial-mesenchymal transition (EMT) or dedifferentiation [6–9]. Tumor budding has been identified as a valuable prognostic marker in many solid tumors, including colorectal cancers, lung cancers, breast cancers and head and neck cancers [10–15]. In our previous studies, we showed that high-intensity tumor budding correlated with lymph node metastasis in patients with TSCC and occult lymph node metastasis in patient with early stage TSCC, indicating that tumor budding may be a promising index for selecting elective neck dissection in patients with cT1/2N0 TSCC [16]. However, the molecular characteristics and the specific mechanisms involved in invasion and metastasis have not been clarified in tumor budding cells of TSCC. In the present study, we aimed to determine the specific miRNA profile of tumor budding cells and investigate the potential underlying mechanisms using laser capture microdissection (LCM) and miRNA arrays. We found that loss of miR-320a promoted invasion and metastasis of tumor budding cells by targeting Suz12 in TSCC. This study established a strong relationship between tumor budding and metastasis triggered by miR-320a, which may be a novel therapeutic target for TSCC patients at risk of metastasis.

## RESULTS

### The miRNA signature and expression of miR-320a in tumor budding cells of TSCC

**Figure 1:**
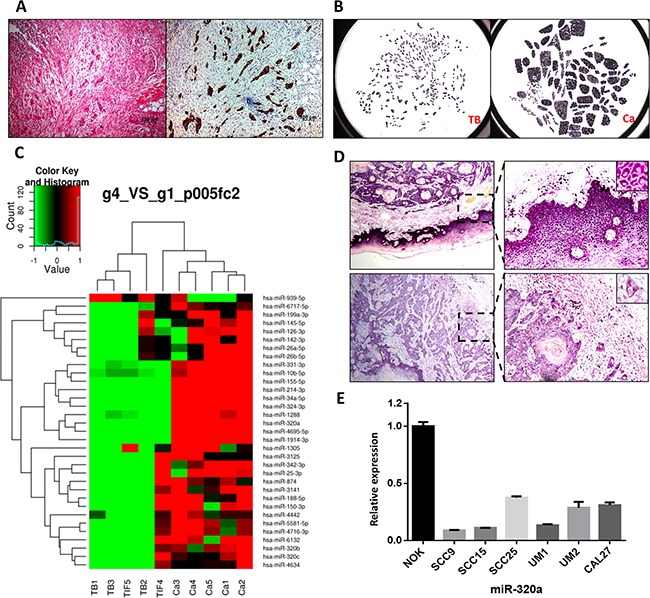
miR-320a is decreased in tumor budding cells of TSCC **A.** HE and pan- cytokeratin staining of tumor budding cells in serial TSCC sections. Original magnification: 100×. **B.** Budding cells (TB) and central tumor tissue (Ca) were captured by LCM. **C.** The different miRNA profiles between tumor budding/the TIF and the central tumor. **D.** The expression of miR-320a in tumor tissues, pericancerous epithelium, and tumor budding cells. **E.** miR-320a expression in TSCC cell lines and NOK cells.

Tumor budding was readily identified in HE-stained samples and further confirmed by immunohistochemistry staining of pan-cytokeratin in serial sections. Budding cells spread into the stroma at the TIF as single cells or small clusters of less than five cancer cells (Figure 1A). These phenotypes indicated that tumor budding cells had a high potential for migration and invasion. As shown in Figure 1B, the budding cells and tumor central tissues were captured and collected using LCM. For the microarray analysis, we found that tumor budding cells had a specific global miRNA signature, with decrease of multiple miRNAs, including miR-320a, b, and c, miR-214, and miR-34a, compared to that of the central tumor tissues (Figure 1C). Because there was little residual total RNA from LCM tissues, qPCR was not used to verify the expression of the miRNAs noted above. Instead, we performed ISH of miR-320a and miR-320b on TSCC samples from the five patients for validation. In every TSCC patient sample, miR-320a was lower in the tumor tissues than that of the adjacent non-cancerous epithelium and much weaker in tumor budding cells at the TIF (Figure 1D). Furthermore, qPCR analysis showed that the expression of miR-320a was decreased in the six TSCC cell lines compared to that of human normal oral keratinocyte (NOK) cells (Figure 1E). However, the expression of miR-320b was not obviously reduced in tumor budding cells, while it was reduced in TSCC cells compared to that of NOK cells (Supplementary Figure S1).

### miR-320a inhibited migration and invasion of TSCC by targeting Suz12 *in vitro*

**Figure 2:**
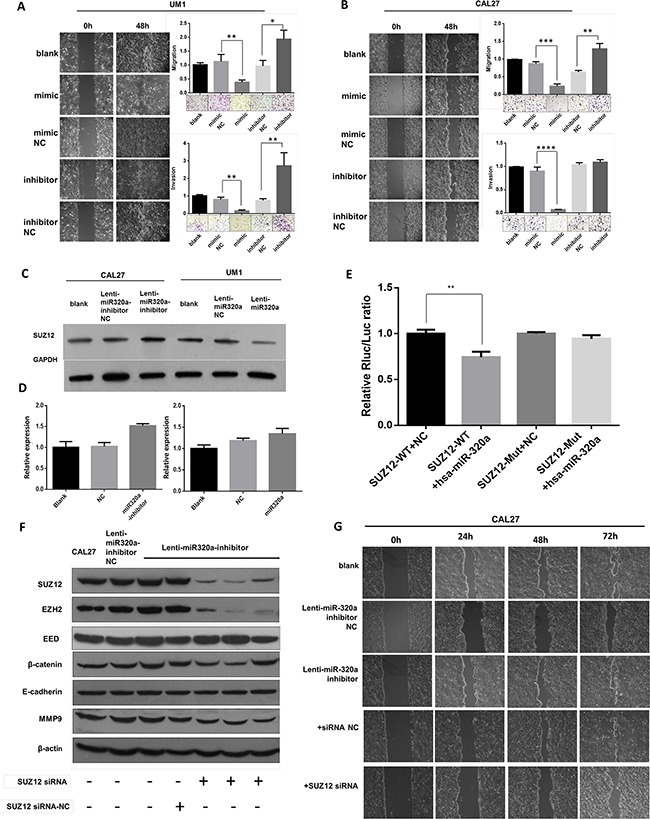
miR-320a suppresses migration and invasion of TSCC cells by targeting Suz12 *in vitro* **A.** Wound healing and transwell assays of UM1 after transient transfection. **B.** Wound healing and transwell assays of Cal27 after transient transfection. **C.** Western blot of Suz12 in stable transfected Cal27 and UM1 cell lines. **D.** Suz12 mRNA in stable transfected Cal27 and UM1 cell lines. **E.** Dual-luciferase reporter gene array. **F.** Western blot assays of Cal27 after co-transfection of Suz12 siRNA. **G.** Wound healing of Cal27 after co-transfection of Suz12 siRNA. *P<0.05, **P<0.01.

To investigate the functional role of miR-320a in migration and invasion of TSCC, we used wound healing and transwell assays. The results showed that miR-320a mimics impaired the cell migration and invasion of UM1 cells, but a miR-320a inhibitor enhanced lateral cell migration and invasion (Figure 2A). Similar results were also observed in Cal27 (Figure 2B) and 1386LN (Supplementary Figure S2) cells. To investigate the mechanisms by which miR-320a suppressed TSCC migration and invasion, we performed a bioinformatics analysis. Target prediction analysis showed that the Suz12 mRNA contains a 3′UTR element that is complementary to miR-320a. More importantly, Suz12 protein and mRNA were both upregulated in Cal27 cells transfected with a lenti-miR320a-inhibitor compared those of the inhibitor-NC and blank groups. In UM1 cells, the Suz12 protein was downregulated when transfected with lenti-miR320a, but no significant change in mRNA was observed (Figure 2C, D). In a dual luciferase reporter assay, the activity of the luciferase reporter gene containing the miR-320a combined site was significantly reduced following transfection by miR-320a mimics (*P*<0.01), and the activity of the luciferase reporter gene containing the mutant miR-320a combined site showed no significant change (*P*>0.05) (Figure 2E). Interestingly, increased expression of EZH2 and β-catenin mediated by miR-320a inhibitors was abolished by Suz12 siRNA (Figure 2F). Wound healing assays showed that the miR-320a knockdown-induced migration was also attenuated (Figure 2G).

### miR-320a suppressed invasion and metastasis of TSCC *in vivo*

**Figure 3:**
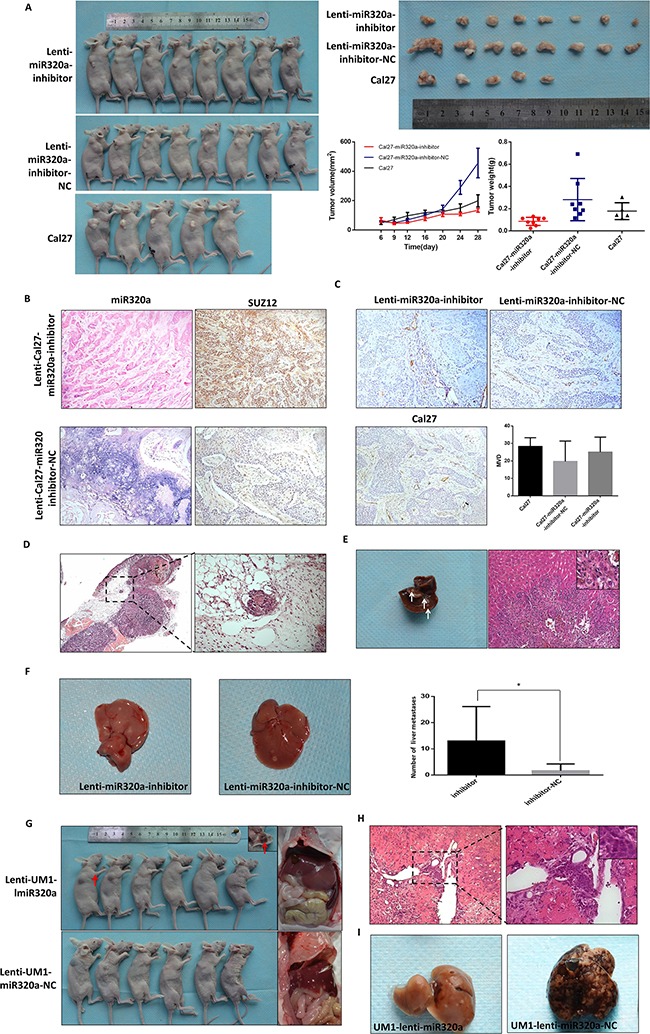
miR-320a suppresses invasion and metastasis of TSCC *in vivo* **A.** The tumor xenografts of Cal27 in subcutaneously injected nude mice. **B.** The expression of miR-320a and SUZ12 in Cal27 tumor xenografts. **C.** The microvessels density (MVD) of Cal27 tumor xenografts labeled by CD31. **D.** Capsular invasion of the Cal27 tumor xenograft cells. **E**, The pathological characteristics of liver metastasis of Cal27 tumor xenografts. **F.** The number of liver metastasis lesion of lenti-Cal27-miR320a-inhibitor by tail vein injection. **G.** The liver metastasis of UM1 subcutaneous injection. Red arrows represent subcutaneous hematoma with fibrous capsule. **H.** The pathological characteristics of liver metastasis of UM1 subcutaneous injection. **I.** The liver metastasis of UM1-miR320a and UM1-miR320a NC cells by tail vein injection. *P<0.05.

To further investigate the effect of miR-320a on invasion and metastasis *in vivo*, we established Cal27 cells with knockdown of miR-320a (lenti-Cal27-miR-320a-inhibitor), UM1 cells overexpressing miR-320a (lenti-UM1-miR-320a) and the respective negative controls and injected these cells into nude mice. All mice injected with Cal27and UM1 cells were sacrificed on day 28 to examine the final tumor growth and organ metastasis. All three groups of Cal27 cell lines formed tumor xenografts in nude mice. The growth curve revealed no tumor volume differences in the three groups (*P*>0.05). The tumor weight of the Cal27-lenti-miR320a-inhibitor group was lighter than the other two groups (*P*=0.0126, *P*=0.0128) (Figure 3A). We hypothesized that the tumor weight increased not only due to the proliferation of cancer cells but also the stroma. Indeed, there were more collagen fibers in the lenti-Cal27-miR320a-inhibitor-NC-derived xenografts than in the lenti-Cal27-miR320a-inhibitor group. ISH showed that the expression of miR-320a in tumor xenografts of the lenti-Cal27-miR320a-inhibitor group was significantly decreased compared to the inhibitor-NC group. Additionally, Suz12 staining in the serial sections was strongly positive, while it was weaker in the inhibitor-NC group (Figure 3B). CD31 staining showed no differences in the microvessels density (MVD) of the three groups (Figure 3C). Capsular invasion was observed in 4/8 of the xenografts from the lenti-Cal27-miR320a-inhibitor group, 1/8 of the xenografts in the Cal27-lenti-miR320a-inhibitor-NC group and 1/5 of the xenografts in the Cal27 blank group (Figure 3D). All infiltrating enveloped cell masses were located in the lateral tumor xenografts, suggesting that they were not formed by the residual cells. Liver metastasis was only detected in lenti-Cal27-miR320a-inhibitor xenografts (1/8) but was not observed in the control and blank groups. The metastatic squamous epithelial cells were located in the central vein of some hepatic lobules, accompanied by hyperplasia of the fibrous connective tissue and infiltration of plasma cells and lymphocytes (Figure 3E). In tail vein model, the number of mice with liver metastases (7/7 nude mice, two dead after injection) and the liver metastases spots (4, 38, 21, 16, 7, 3, 2) of lenti-Cal27-miR320a-inhibitor group were both more than negative control group (3/8 nude mice, one dead after injection; 2, 7, 4 metastases spots), there were statistically significant differences in the number of metastasis spots (*P*=0.0317) (Figure 3F). These results suggested that knockdown of miR-320a could enhance the invasive capacity of Cal27 cells.

UM1 cell lines did not form subcutaneous tumor xenografts but instead formed hematomas (Figure 3G). However, more liver metastases were observed in the lenti-UM1-miR320a-NC group (6/6) than the lenti-UM1-miR320a group (2/6). HE staining showed that the metastatic squamous epithelial cells had tight junctions, and the surrounding liver tissues were degenerated (Figure 3H). Liver metastases were detected in 33.3% mice with the lenti-UM1-miR-320a cells (2/6) and 100% mice with the lenti-UM1-miR-320a-NC cells (6/6) (Figure 3I) of a tail vein injection model. These results strongly suggested that upregulation of miR-320a could suppress tumor metastasis in nude mice.

### Tumor budding, miR-320 and Suz12 in TSCC tissue samples

**Figure 4:**
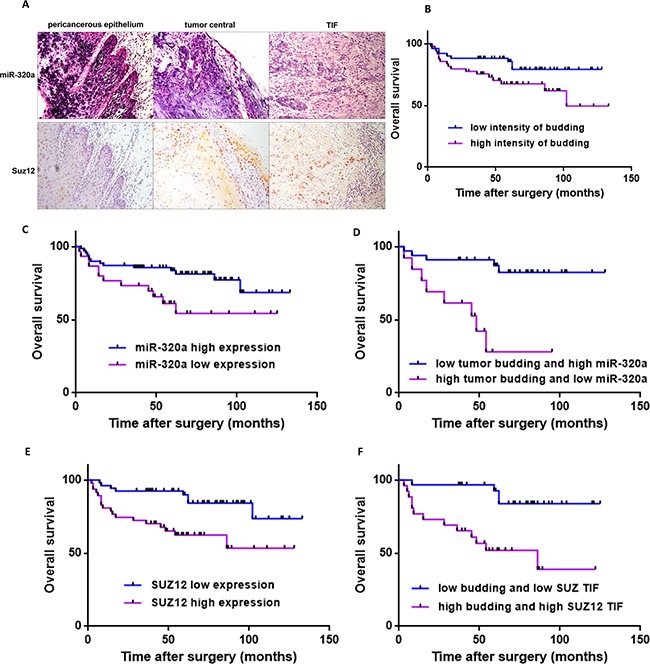
Cumulative overall survival curves after surgery in TSCC patients **A.** The expression of miR-320a and Suz12 in pericancerous epithelium, the central tumor and the TIF in samples from one TSCC patient. Original magnification: 200×. **B.** Kaplan-Meier curves for the overall survival of patients with low-intensity (*n*=51) versus high-intensity (*n*=49) budding. **C.** Kaplan-Meier curves for the overall survival of patients with low expression of miR-320a (*n*=30) versus high expression (*n*=70). **D.** Kaplan-Meier curves for the overall survival of patients with low-intensity budding and high miR-320a (*n*=33) versus high-intensity budding and low miR-320a (*n*=13). **E.** Kaplan-Meier curves for the overall survival of patients with high Suz12 expression at the TIF (*n*=47) versus low expression (*n*=53). **F.** Kaplan-Meier curves for the overall survival of patients with low-intensity budding and low Suz12 (*n*=30) versus high-intensity budding and high Suz12 (*n*=26).

To further confirm the role of tumor budding, miR-320 and Suz12 in TSCC, we performed a clinicopathological analysis of 100 patients, of which 48 were male and 52 were female. The average age was 53.68, and the median age was 54. The clinicopathological parameters of the 100 TSCC cases are summarized in Supplementary Table S1. The expression of miR-320a was lower in tumor tissues than the pericancerous epithelium and was very weak in tumor budding cells at the TIF. Suz12 was predominantly located in the nucleus and was upregulated in the central tumor and TIF; thus it showed a negative correlation with miR-320a in the TSCC slices (Figure 4A). Forty-nine patients (49%) were classified into the high-intensity budding group, and the remaining 51 patients were in the low group. Of the 51 low-grade budding cases, no tumor budding was observed in 11 cases. Consequently, the frequency of buds in TSCC in this study was 89%. At the time of the last follow-up, 9 of 51 patients with low-grade budding and 17 of 49 patients with high-grade budding had died. The cumulative survival rate for the low-grade group was significantly higher than that of the high-grade group (*P*<0.05, log rank test; Figure 4B). The survival of patients with high expression of miR-320a was higher than that of patients with low expression (*P*=0.0285, log rank test; Figure 4C). Moreover, the patients with low tumor budding and high miR-320a had a substantially higher survival rate than the patients with high tumor budding and low miR-320a expression (*P*=0.0002, log rank test; Figure 4D). The survival of patients with low expression of Suz12 at the TIF was higher than that of patients with high expression (*P* =0.0029, log rank test; Figure 4E). Furthermore, the survival rate of patients with low budding and low Suz12 at the TIF was higher than that of the patients with high budding and high Suz12 at the TIF (*P*=0.0007, log rank test; Figure 4F).

Correlations were tested among tumor budding, miR-320a, Suz12 and clinical parameters of the TSCC cases (Table 1). The results showed an association of lymphoid infiltrate with T classification (*P*<0.005) and invasive pattern (*P*<0.05). Invasive depth was related to T classification (*P*<0.05). As expected, correlations were also observed between the intensity of tumor budding and lymph node metastasis (*P*<0.01). Interestingly, a correlation between age and tumor budding was also observed (*p*<0.01). Suz12 was associated with tumor relapse (*P*<0.05). Consistent with the observed TSCC slices, Spearman's correlation demonstrated that miR-320a and Suz12 were negatively correlated (*P*<0.05), especially at the TIF (*P*<0.05).

**Table 1:** Correlations among clinical and histopathological features of primary TSCC^a^

Gender	Age (years)	T	Lymph node metastasis	Pathologic differentiation	Invasive pattern*	Lymphoid infiltrate	Invasive depth	Tumor budding	Relapse	SUZ12 TIF	SUZ12 total	miR-320a TIF	miR-320a total
**Gender**	−0.147 (0.143)	0.070 (0.492)	0.025 (0.828)	−0.058 (0.564)	−0.093 (0.358)	0.155 (0.123)	0.085 (0.402)	0.059 (0.558)	−0.107 (0.291)	−0.143 (0.156)	0.080 (0.428)	0.095 (0.345)	0.017 (0.863)
**Age(years)**		−0.164 (0.103)	0.733 (0.522)	0.705 (0.458)	0.708 (0.441)	−0.026 (0.800)	−0.119 (0.238)	0.273 (0.006)**	0.086 (0.397)	0.079 (0.434)	0.043 (0.669)	−0.032 (0.756)	0.043 (0.670)
**T classification**			0.136 (0.233)	0.097 (0.335)	0.017 (0.830)	0.292 (0.003) **	0.202 (0.044)*	−0.077 (0.449)	0.091 (0.369)	0.082 (0.417)	0.045 (0.665)	0.043 (0.670)	−0.078 (0.442)
**Lymph node metastasis**				−0.144 (0.205)	0.012 (0.916)	0.129 (0.257)	0.169 (0.136)	0.224 (0.047)*	0.070 (0.538)	0.206 (0.068)	0.044 (0.703)	0.089 (0.433)	0.163 (0.152)
**Pathologic differentiation**					−0.14 (0.164)	−0051 (0.613)	0.060 (0.550)	0.142 (0.158)	0.064 (0.528)	0.041 (0.684)	0.086 (0.396)	0.054 (0.596)	0.122 (0.228)
**Invasive pattern^b^**						0.216 (0.031)*	0.058 (0.567)	−0.077 (0.444)	−0.144 (0.257)	−0.191 (0.057)	−0.021 (0.838)	0.122 (0.228)	−0.005 (0.946)
**Lymphoid infiltrate**							0.046 (0.647)	0.057 (0.547)	0.027 (0.786)	−0.077 (0.448)	0.000 (1.000)	0.194 (0.053)	−0.027 (0.790)
**Invasive depth**								0.142 (0.160)	−0.084 (0.407)	0.072 (0.477)	0.022 (0.828)	0.065 (0.518)	0.063 (0.537)
**Tumor budding**									0.123 (0.222)	0.119 (0.238)	0.100 (0.322)	0.118 (0.241)	0.074 (0.463)
**Relapse**										0.213 (0.034)*	0.118 (0.244)	−0.001 (0.994)	0.009 (0.933)
**SUZ12 TIF**											−0.301 (0.002)*	−0.251 (0.012)*	−0.171 (0.090)
**SUZ12 total**												0.101 (0.317)	0.218 (0.029)*
**miR-320a TIF**													0.0348 (0.000)*
**miR-320a total**													

a Spearman correlation coefficients were presented

*P<0.05, **P<0.01

b Type1: broad pushing manner

Type2: broad pushing ‘fingers’ or separate large tumor islands

Type3: invasive islands of tumor greater than 15 cells per island

Type4: invasive tumor islands smaller than 15 cells per island

Univariate analysis demonstrated that invasive depth, tumor budding, expression of Suz12 at the TIF, T classification, lymph node metastasis and overall expression of miR-320a were statistically significant predictors of prognosis. Multivariate analysis was performed using these pathological factors as covariates, and T classification, lymph node metastasis and miR-320a overall expression were identified as significant independent prognostic factors (Table 2).

**Table 2:** Cox regression models of patients with TSCC for clinical and pathologic factors

Characteristic	Subcharacteristic	Univariate Analysis			Multivariate Analysis		
		HR	95 % CI	*P*	HR	95 % CI	*P*
**Gender**	Female	1					
	Male	2.206	0.983-4.950	0.055			
**Age(years)**	<60	1					
	≥60	1.008	0.977-1.040	0.611			
**Pathologic differentiation**	Well	1					
	Moderately/Poorly	1.153	0.513-2.588	0.731			
**Lymphoid infiltrate**	Dense and continuous	1					
	Discontimuous/limited	1.718	0.792-3.726	0.171			
**Invasive pattern^a^**	Type1/2	1					
	Type3/4	1.618	0.680-3.851	0.277			
**Local relapse**	No	1					
	Yes	2.081	0.622-6.964	0.234			
**SUZ12 total**	Low	1					
	High	0.725	0.333-1.578	0.418			
**miR-320a TIF**	Low	1					
	High	0.649	0.288-1.461	0.296			
**Invasive depth**	≤4mm	1					
	>4mm	3.616	1.085-12.058	**0.036**			
**Tumor budding**	Low grade	1					
	High grade	2.228	0.991-5.008	**0.046**			
**SUZ12 TIF**	Low	1					
	High	3.311	1.432-7.658	**0.005**			
**T classification**	T1/2	1			1		
	T3/4	3.752	1.724-8.164	**0.001**	2.423	1.074-5.464	**0.033**
**Lymph node metastasis**	Negative	1			1		
	Positive	3.722	1.654-8.373	**0.001**	3.939	1.704-9.104	**0.001**
**miR-320a overall**	Low	1			1		
	High	0.433	0.200-0.939	**0.034**	0.367	0.161-0.837	**0.017**

The status of lymph node metastasis was available for only 79 patients

a Type1: broad pushing manner

Type2: broad pushing ‘fingers’ or separate large tumor islands

Type3: invasive islands of tumor greater than 15 cells per island

Type4: invasive tumor islands smaller than 15 cells per island

## DISCUSSION

Tumor budding is a histopathological finding that has been suggested to be an important prognostic factor in many types of cancers [17, 18, 19, 20, 21]. In previous studies, we found that high intensity of tumor budding was correlated with EMT in TSCC patients [13] and occult lymph node metastasis in patients with clinical early stage TSCC [16]. Jensen et al. found that tumor budding cells exhibited particular EMT molecular signatures in oral squamous cell carcinoma, which were characterized by downregulation of the miR-200 family and activation of TGFβ signaling [22]. However, the molecular characteristics of tumor budding cells and the underlying mechanisms governing this phenotype are still poorly understood in TSCC. In this study, we employed LCM and miRNA arrays to explore the miRNA profile of tumor budding cells and investigate their potential mechanism. A new miRNA expression profile was identified, and 33 miRNAs were found to be down-regulated in budding cells compared to the more cohesive central tumor cells. Interestingly, the members of miR-320 family (miR-320a, b and c) were significantly decreased in tumor budding cells but not the miR-200 family, and the loss of miR-320a was further confirmed with ISH and qPCR performed in patient tissue samples and 6 TSCC cell lines compared with NOK cells. More interestingly, several studies suggested that miR-320 family members could function as tumor suppressors in several cancers, including prostate cancer, colorectal cancer, colon cancer and cervical cancer [23, 24–27]. These findings indicated that miR-320a may have a pivotal role in tumor budding-mediated migration and invasion.

To verify the effect of miR-320a on migration and invasion, miR-320a mimics and inhibitors were transfected into TSCC cells, and wound-healing and transwell assays showed that miR-320a could suppress migration and invasion in TSCC cells. Similar results were also observed in hepatocellular carcinoma and cervical cancer [27,28]. To further investigate the mechanism of miR-320a in migration and invasion of TSCC, we performed a bioinformatics analysis, and targets of miR-320a were predicted. GO analysis showed that the miR-320a target genes were predominantly involved in energy metabolism, biosynthesis, tumor genesis and cell migration. KEGG pathway analysis showed that the target genes of miR-320a participated in many pathways, including colorectal cancer development, pathways in cancer, and the Wnt signaling pathway. Based on the seed sequence of miR-320a, the target prediction program indicated that Suz12 may be a direct target of miR-320a. Thus, we further confirmed that Suz12 is a direct target of miR-320a in TSCC using WB, IF and dual luciferase reporter assays. a of Suz12 could partially reverse the miR-320a inhibitor-mediated migration. These results confirmed that Suz12 was involved in miR-320a-mediated suppression of migration and invasion. Suz12 is a key component of the polycomb repressive complex 2 (PRC2), which has been confirmed to promote invasion and metastasis by epigenetically silencing metastasis suppressors in many malignant tumors [29–32]. In a previous study, we also confirmed that PRC2 could suppress E-cadherin expression to promote invasion and metastasis of TSCC [33]. On the basis of these results, we concluded that loss of miR-320a could suppress migration and invasion by targeting Suz12 in TSCC cells.

To further determine the effect of miR-320a on TSCC invasion and metastasis *in vivo*, we established tumor xenograft models in nude mice through subcutaneous and vein injection using Cal27 (high expression of endogenous miR-320a) and UM1 (low expression of endogenous miR-320a) cell lines. In Cal27 cell lines, knockdown of miR-320a significantly promoted invasion and liver metastasis of TSCC in both subcutaneous and vein injection models. However, subcutaneous xenografts tumors were not formed by UM1 cells with and without stable overexpression of miR-320a. Interestingly, liver metastases were dramatically decreased in UM1 cells with knock-in miR-320a compared to cells without knock-in miR-320a. A strong inverse correlation was also observed between miR-320a and Suz12 as determined by IHC and ISH in xenograft tumor samples. These findings are consistent with the *in vitro* observations.

Finally, to assess the clinical significance and prognostic value of tumor budding, miR-320a and Suz12 in TSCC patients, we performed ISH and IHC in another patient cohort with 100 TSCC patients. The high intensity of tumor budding was positively correlated with lymph node metastasis in TSCC patients. Similar results were also observed in our previous studies in different TSCC patient cohorts and other cancer types, which indicated that tumor budding can serve as a strong pathological indicator of lymph node metastasis [13, 16, 34–36]. The expression of miR-320a was also inversely correlated with Suz12 expression, which confirmed that Suz12 was targeted by miR-320a. Furthermore, we found that high intensity of tumor budding, decreased expression of miR-320a and increased expression of Suz12 in TSCC were strong predictors of decreased overall survival. A dramatically reduced survival rate was observed in patients with high intensity of tumor budding and decreased expression of miR-320a compared with patients with low-intensity tumor budding and increased expression of miR-320a. Thus, tumor budding and miR-320a expression are potential predictors of the prognosis of TSCC patients. The examination of tumor budding and miR-320a expression by routine HE and ISH staining, therefore, may be used as an effective tool to identify patients with TSCC at increased risk of tumor progression and metastasis or patients with cT1/2N0 TSCC for elective neck dissection. These findings indicate a critical role of tumor budding and miR-320a in the invasion and metastasis of TSCC.

Taken together, our present study identified the miRNA expression signature of tumor budding in TSCC. Our results suggest that miR-320a has a critical role in the acquisition of an aggressive and/or metastatic phenotype in tumor budding cells of TSCC. Furthermore, miR-320a-mediated repression of invasion and metastasis is achieved, at least in part, by down-regulating Suz12 expression. Therefore, miR-320a and tumor budding may be the new biomarkers and therapeutic targets for the treatment of TSCC metastases.

## MATERIALS AND METHODS

### Patients

Two patient cohorts with TSCC were enrolled in this study. Cohort 1 with five TSCC patients underwent resection of the primary tumor and neck dissection at the Hospital of Stomatology, Sun Yat-sen University between January 2013 and May 2013. The TSCC tissue samples were prepared for laser capture microdissection (LCM) and miRNA microarrays. Cohort 2 consisted of 100 archived TSCC samples, which were retrieved and prepared for clinicopathological analysis and validation. The patients in this cohort received resection of the primary tumor with or without neck dissection between January 2001 and December 2010 at the Hospital of Stomatology or the first Affiliated Hospital, Sun Yat-sen University. All patients received no radiotherapy or chemotherapy before surgery. The tumor stage was classified according to the TNM system by UICC. The survival data were collected by consulting the medical records or telephone follow-up. Survival time was calculated from the date of the major surgery to the last follow-up (between December 2013 and January 2014) or death. This study was approved by the ethical committee of Sun Yat-Sen University and Guanghua School of Stomatology.

### LCM, microRNA array and bioinformatics analysis

For patient cohort 1, 10 μm thick primary tumor samples were obtained and stained with HE. Then, tumor budding cells and tumor central tissues were captured from PEN membrane slides by laser capture microdissection (ArcturusXT™ Laser Capture Microdissection Systems, Thermo Fisher) as previously described [37]. Each tissue sample from the same site of one patient was pooled to create one biological sample. Total RNA was extracted using TRIzol Reagent (Life Technologies) and further purified by an RNeasy Micro kit (Qiagen, GmBH) and an RNase-Free DNase Set (Qiagen, GmBH). Total RNA was amplified, labeled and purified by an Affymetrix WT PLUS Reagent Kit (Affymetrix) according to the manufacturer's instructions to obtain biotin-labeled cDNA. Arrays were scanned with an Affymetrix GeneChip® Scanner 3000 (Affymetrix). Command Console Software (Affymetrix) was used to control the scanner and summarize probe cell intensity data (CEL file generation) with default settings. The raw data were normalized by an Expression Console. miRWalk was used to predict the miRNA target genes. The DAVID database (Database for Annotation, Visualization, and Integrated Discovery) was used to analyze the biological processes and signaling pathways of miRNA by GO and Pathway analyses.

### *In situ* hybridization, immunohistochemistry and clinicopathological analysis

Double-DIG-labeled LNA™ microRNA probes (1:1250, Exiqon) were used for miRNA-320a amplification analysis following the manufacturer's instructions of the microRNA ISH Optimization Kit for FFPE (Exiqon). Immunohistochemical staining was performed with a Suz12 antibody (1:100, Abcam) and pan-cytokeratin (1:1000, BD). Then 3, 3′-diaminobenzidine (DAB, Sigma-Aldrich) was used for visualization. Blank controls were included by substituting non-immune serum for primary antibodies.

All specimens were independently assessed by the two pathologists blinded to the clinical data. The pathological differentiation was classified according to the WHO classification. The mode invasion and lymphoid infiltrate were determined using the classification of Brandwein-Gensler et al. [38]. Lymph node metastasis and tumor relapse were also confirmed microscopically by reviewing the specimens from lymph node dissection and regional recurrence of tissue from the tongue. Tumor budding was identified in HE-stained samples and further confirmed by immunohistochemistry staining of pan-cytokeratin in serial sections as previously described [13]. Tumor depth was measured from the surface of the mucosa to the deepest portion of the tumor [39]. The score of miRNA-320a or Suz12 staining was determined by the proportion of positively stained tumor cells combined with the intensity of staining. miR-320a expression in the overall slice or at the TIF was evaluated. For the miR-320a score, the proportion of positively stained cells was graded as follows: 0 (positive cells≤10%) or 1 (positive cells >10%). The intensity of staining was recorded as follows: 0 (no staining), 1 (weak), 2 (moderate), or 3 (strong). The score of each slice was graded as the staining index (SI) = proportion of positively stained cells×staining intensity [40]. SI (miR-320a) >1 was the high-grade expression group, and IS≤1 was the low-grade expression group. For Suz12 grading, the proportion of positively stained cells was graded as 0 (no staining), 1 (positive cells≤10%), 2 (10%< positive cells≤50%), 3 (50%< positive cells≤80%), or 4 (80%< positive cells). The intensity of staining was evaluated as miR-320a. SI (Suz12) >8 was defined as high-grade expression, and SI≤8 was defined as low-grade expression.

### Cell lines, transient transfection and lentiviral transfection

TSCC cells were cultivated and inoculated as previously described [41]. miR-320a mimics (50 nM, RiboBio), miR-320a inhibitor (100nM, RiboBio), Suz12 siRNA (100nM, RiboBio) and their negative controls were transfected into TSCC cells using Lipofectamine (Lipofectamine RNA iMAX Transfection Reagent, Thermo Fisher) for downstream arrays. The fluorescence detection showed the transfected efficiency was more than 90%. RT-qPCR showed that miR-320a increased more than 3000 times transfected by mimics, and decreased about 1 times transfected by inhibitor. UM1 cells were infected by lentiviruses (pEZX-MR03 vector) containing a miR-320a precursor (miExpress™ Precursor miRNA Expression Clone, HmiR0129-MR03, GeneCopoeia) or a negative clone sequence. The endogenous miR-320a inhibitor vector (miArrest™ miRNA inhibitor Expression Clone, HmiR-AN0405-AM03, GeneCopoeia) and negative control were transfected into Cal27 cells by lentivectors. The lentiviral packaging plasmids were purchased from GeneCopoeia. Packaging of lentiviral vector into pseudoviral particles and delivery of packaged lentivectors into UM1 or Cal27 cells were performed according to the manufacturer's instructions. The fluorescence detection showed the transfected cells with green (UM1) or red (Cal27) fluorescence. RT-qPCR showed that miR-320a in stably transfected cell lines UM1 was up to about 115 times, and declined in cal27.

### Wound healing and transwell assays

Wound healing assays were performed when the cells reached 90% confluence in six-well plates. Wounds were made with a pipette tip in confluent monolayers. At 0 h and 48 h, the plates were examined and photographed microscopically. Each cell condition was assayed in triplicate wells.

The transfected UM1 or Cal27 cells were seeded into the upper chambers with or without Matrigel™ Basement Membrane Matrix for the invasion or migration assays (BD Biosciences). Culture medium with 10% fetal bovine serum was added to the lower wells to stimulate cell invasion. TSCC cells were allowed to migrate for 24 h. Then, cells in upper chambers were removed. The membranes of the upper chamber were stained with crystal violet (MedChemExpress, Princeton, USA). The numbers of invaded cells were assessed using a light microscope (ZEISS, German) and counted in three fields (magnification: 200). Independent-samples T test was performed by SPSS software (19.0, IBM).

### Real-time reverse-transcriptase (RT)-PCR and western blot

Total RNA was extracted via TRIzol (Life Technologies, Carlsbad, CA) or an isolation kit (mirVana™ miRNA Isolation Kit, Ambion, USA). Reverse transcription was performed from total RNA using a Transcriptor First Strand cDNA Synthesis Kit (Roche) following the manufacturer's instructions. The qPCR primer of Suz12 and 18S rRNA was purchased from TaKaRa (Ruizhen). Bulge-loop™ miRNA qRT-PCR Primer Sets (one RT primer and a pair of qPCR primers for each set) specific to miR-320a and U6 were designed by RiboBio. The qPCR reactions were performed on a Light Cycler 480 system (Roche) with SYBR Green I Master reagents (Roche).

Western blots were performed as described previously [41] using antibodies specific to Suz12 (1:100, Abcam), EZH2 (1:2000, Origene), EED (1:2000, (Millipore), β-catenin (1:7500, Abcam), E-cadherin (1:1000, Cell Signaling Technology), and MMP-9 (1:2000, Proteintech). ImageJ software was used to analyze the expression of target proteins by comparison to GAPDH (1:5000, HC301) or H3 (1:2000, Cell Signaling Technology).

### Dual-luciferase reporter assay

The pmiR-RB-REPORT™ vector was designed to detect the effect of miR-320a on Suz12. DH5α competent cells were co-transfected with Suz12 (human) 3′UTR-luciferase reporter plasmid or its mutant. Then, cells were co-transfected with miR-320a mimics or mimic NC. Luciferase assays were performed at 48 h after transfection using a Dual-Luciferase Reporter Assay System (Promega, Madison, WI). *Renilla* luciferase activity was normalized to firefly luciferase activity and compared between groups. Triplicate transfections were tested.

### TSCC tumor model in nude mice

The animal experiments were approved by the experimental animal ethics committee of Sun Yat-Sen University. Female BALB/c-nu mice of 4 to 6 weeks were divided into the blank control (Cal27 or UM1 cells without any treatment), the stable transfected cell (lenti-Cal27-miR320a-inhibitor or lenti-UM1-miR320a), or the transfected control (lenti-Cal27-miR320a-inhibitor-NC or lenti-UM1-miR320a-NC) groups for subcutaneous or vein injection. In the subcutaneous model, 200 μl cell suspensions containing 5,000,000 cells were injected into the subcutaneous tissue at unilateral back adjacent to the middle axillary. The tumor volume was measured twice a week after inoculation to generate the tumor growth curve. Tumor volume was calculated by the following formula: tumor volume (TV)=(length×width×width)/2 [42]. The mice were sacrificed on day 28 after inoculation. Tumor volume and weight were recorded. The tumor xenografts were fixed and prepared for pathological examinations. In the vein injection model, 400 μl cell suspensions containing 4,000,000 cells were injected into mice. The mice were sacrificed on day 28 after injection. The lungs and livers were dissected, observed and fixed for pathology analysis.

### Statistical analyses

Spearman's correlation coefficient was used to assess correlations between two histopathological parameters. Kaplan-Meier plots were generated to present the survival outcomes by GraphPad software (Prism 6). Both univariate and multivariate analyses of several clinical and pathological factors were performed by Cox regression using Forward method. The statistical calculations were performed by SPSS software (19.0, IBM). For all statistical analyses, *P*-values of <0.05 (two tailed) were considered to be as statistically significant.

## SUPPLEMENTARY FIGURES AND TABLES


