# Supplementary material for: Decreased miR-320a promotes invasion and metastasis of tumor budding cells in tongue squamous cell carcinoma
Source: Oncotarget. 2016 Aug 25;7(40):65744–57. doi: 10.18632/oncotarget.11612 (PMC5323189; doi:10.18632/oncotarget.11612)
Supplement: Supplementary file 1 [file oncotarget-07-65744-s001.pdf]

Decreased miR-320a promotes invasion and metastasis of tumor budding cells in tongue squamous cell carcinoma

SUPPLEMENTARY FIGURES AND TABLE

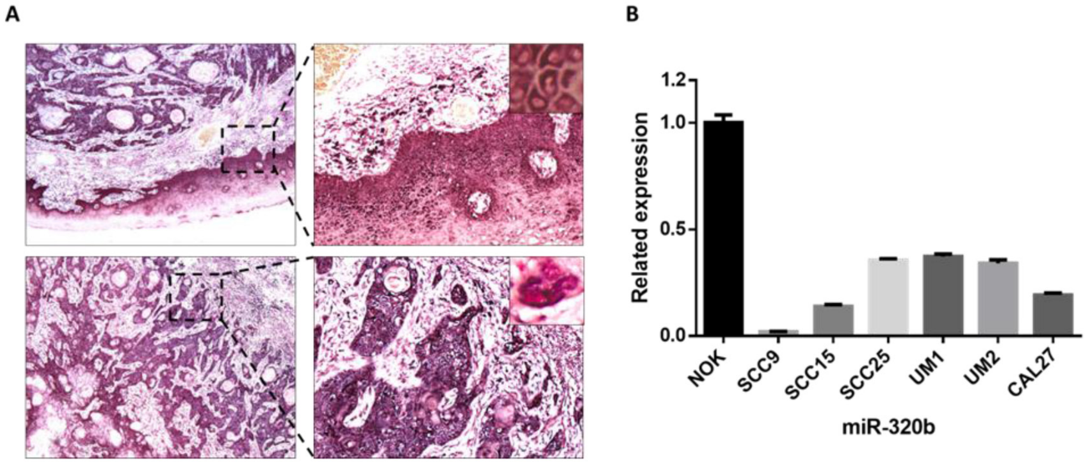

Supplementary Figure S1: miR-320b in TSCC. A. The expression of miR-320b in tumor tissue, ericancerous epithelium, and tumor budding. **B.** miR-320b expression in TSCC cell lines and NOK.

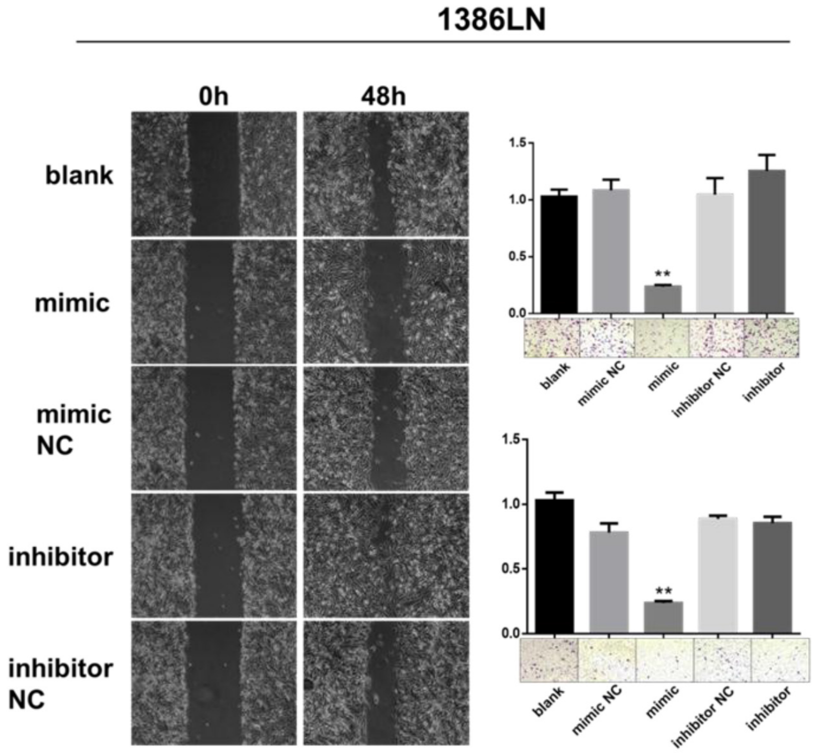

Supplementary Figure S2: miR-320a suppresses migration and invasion of 1386LN. Wound healing and transwell assay of 1386LN after transient transfection.

Supplementary Table S1: Clinicopathologic features of 100 TSCC patients

Characteristic	Subcharacteristic	*n*
**Gender**	Male	48
	Female	52
**Age(years)**	<60	66
	≥60	34
**T classification**	T1/T2	72
	T3/T4	28
**Lymph node metastasis**	Negative	28
	Positive	51
**Pathologic differentiation**	Well	68
	Moderately/ Poorly	32
**Invasive pattern[a]**	Type1/Type2	37
	Type3/Type4	63
**Lymphoid infiltrate**	Dense and continuous	62
	Discontinuous/ Limited	38
**Invasive depth**	≤4mm	29
	>4mm	71
**Tumor budding**	Low	51
	High	49
**Relapse**	No	93
	Yes	7
**SUZ12 overall**	Low	50
	High	50
**SUZ12 TIF**	Low	53
	High	47
**miR0320a overall**	Low	30
	High	70
**miR-320a TIF**	Low	57
	High	43

Lymph node metastasis was known for only 79 patients
a Type1: broad pushing manner
Type2: broad pushing ‘fingers’ or separate large tumor islands
Type3: invasive islands of tumor greater than 15 cells per island
Type4: invasive tumor islands smaller than 15 cells per island
